# Templated green synthesis of plasmonic silver nanoparticles in onion epidermal cells suitable for surface-enhanced Raman and hyper-Raman scattering

**DOI:** 10.3762/bjnano.7.75

**Published:** 2016-06-09

**Authors:** Marta Espina Palanco, Klaus Bo Mogensen, Marina Gühlke, Zsuzsanna Heiner, Janina Kneipp, Katrin Kneipp

**Affiliations:** 1 Danmarks Tekniske Universitet DTU, Department of Physics, 2800 Kgs. Lyngby, Denmark; 2 Danmarks Tekniske Universitet DTU, Department of Micro- and Nanotechnology. 2800 Kgs. Lyngby, Denmark, present affiliation: Philips Biocell, Gydevang 42, 3450 Allerød, Denmark; 3 Humboldt Universität zu Berlin, 12489 Berlin, Germany

**Keywords:** biotemplates, green preparation, onion, plasmonic nanoparticles, surface-enhanced Raman scattering, surface-enhanced hyper-Raman scattering

## Abstract

We report fast and simple green synthesis of plasmonic silver nanoparticles in the epidermal cells of onions after incubation with AgNO_3_ solution. The biological environment supports the generation of silver nanostructures in two ways. The plant tissue delivers reducing chemicals for the initial formation of small silver clusters and their following conversion to plasmonic particles. Additionally, the natural morphological structures of the onion layers, in particular the extracellular matrix provides a biological template for the growth of plasmonic nanostructures. This is indicated by red glowing images of extracellular spaces in dark field microscopy of onion layers a few hours after AgNO_3_ exposure due to the formation of silver nanoparticles. Silver nanostructures generated in the extracellular space of onion layers and within the epidermal cell walls can serve as enhancing plasmonic structures for one- and two-photon-excited spectroscopy such as surface enhanced Raman scattering (SERS) and surface enhanced hyper-Raman scattering (SEHRS). Our studies demonstrate a templated green preparation of enhancing plasmonic nanoparticles and suggest a new route to deliver silver nanoparticles as basic building blocks of plasmonic nanosensors to plants by the uptake of solutions of metal salts.

## Introduction

Nanostructures made from metals, such as silver, gold, aluminium or palladium in various sizes and shapes attract growing attention because of their interesting properties and broad applications in many different fields of science, technology and medicine [1]. Particularly exciting applications of metal nanostructures exploit the resonant interaction of light with the collective oscillations of the free electrons, so-called surface plasmons. These resonances can give rise to strongly enhanced and highly confined local optical fields in the vicinity of metal nanostructures. Plasmonic field enhancement enables optical and spectroscopic measurements at unprecedented sensitivity and spatial resolution [2]. For chemical analysis, Raman spectroscopy performed in enhanced local fields allows for the detection and structural characterization of single molecules [3]. Beside these advances in sensing and probing, plasmonics has the potential to revolutionize almost all photonic technologies [4].

Numerous technologically and medically relevant applications and particularly also the rapid development of plasmonics as a field of research generate a strong interest in manufacturing metal nanostructures of well-defined morphologies that support plasmon resonances at very different energies within the entire optical spectral range. Moreover, metal nanostructures should be prepared in simple and fast, cheap and also environmentally friendly processes.

Very popular preparation methods of silver and gold nanostructures are based on bottom-up processes, where nanoparticles, are built from smaller structures such as metal ions. Sodium citrate and sodium borohydride are very common reducing chemicals for metal salts [5–6], but also more eco-friendly compounds such as glucose or starch can be used for the preparation of metal nanoparticles [7–8].

Overall, various reducing and stabilization agents as well as variations in experimental conditions during the preparation process, such as the influence of light or temperature , as well as sonochemical preparation routes allow for the synthesis of silver and gold nanoparticles of various sizes and shapes [9–10]. Additionally, appropriate templates for the growing process can define and control size, shape and assembling of nanostructures [9,11–12].

During the last decade, so-called “green synthesis” came into the focus of interest, since many molecules typically available in biological living matter have the capability to reduce silver and gold salts. It has been demonstrated that plants and also microorganisms such as algae, fungi, yeasts, and bacteria provide chemicals suitable for the preparation of metal nanoparticles [13–14]. For example, different parts of plants contain polysaccharides, phenolics, or flavonoids, to mention only a few compounds, which could serve as reducing and also stabilizing agents. The preparation of silver and gold nanoparticles using many very different pre-treated plant materials, such as extracts collected from leafs or vegetables and fruits has been demonstrated in numerous publications [15–19]. The diversity of bioorganic molecules available in plants provides many combinations of reducing and stabilizing agents. This gives rise to a broad variety of parameters in the green preparation process, resulting in metal nanoparticles of different sizes and shapes.

While pre-treated plant materials such as extract and juice have been used in former studies [14–15,18–20]. In this article, we demonstrate and discuss the green preparation of plasmonic silver nanoparticles in intact onion epidermal cells after incubation with AgNO_3_ solution. The onions deliver reducing and stabilizing chemicals, while the histological structure of the onion layer, in particular the cell walls and the extracellular space they surround provide a biological template for the growth process of plasmonic silver structures. In our experimental study, we exploit luminescence spectroscopy and dark-field microscopy for probing the formation of metal nanostructures in situ in the onion tissue. Local optical fields related to the plasmonic nanostructures are probed by surface-enhanced Raman scattering (SERS) and two-photon-excited analogous surface-enhanced hyper-Raman scattering (SEHRS) [21–22]. While SERS signals scale with the local optical field strengths by 10^4^, SEHRS signals have a scaling factor of 10^6^. This high non-linearity makes SEHRS a very sensitive method to probe spatial variations in local fields and to localize plasmonic nanostructures, surpassing also SERS. Here we compare SEHRS images and bright field microscopy of the onion cell layers. Additionally, our SERS and SEHRS experiments give evidence of the capability of the “green” silver nanostructures to enhance one- and two-photon-excited optical processes.

## Experimental

### Sample preparation

A single cell layer of a red onion, purchased from the supermarket, was peeled from fresh vegetables and pieces of about 1 cm^2^ were placed in an aqueous silver nitrate solution (10^−3^ M concentration from 99.9% pure AgNO_3_, Sigma-Aldrich Denmark A/S)*.* After 20 h of incubation at room temperature and in darkness, the pieces were removed, rinsed with tap water and placed on a glass slide to dry for several hours, also in darkness. After drying, the samples were ready for optical experiments. In order to also explore the potential formation of gold nanostructures in onion layers and for a comparison between green preparation of silver and gold nanostructures, the same sample preparation was applied using aqueous chloroauric acid, HAuCl_4_ (10^−3^ M, Sigma-Aldrich Denmark A/S) instead of AgNO_3_.

### Luminescence measurements and dark-field microscopy

Luminescence spectra and images were measured through a 100× oil immersion objective (Leica DMLM microscope) using a laser diode (473 nm, ca. 20 mW) for excitation and equipped with a λ = 520 nm long-wave pass filter for emission. Dark Field microscopy was carried out using a Nikon eclipse LN200N microscope with 50 W halogen lamp for illuminating the sample. The spectra were collected using a fiber-coupled spectrum analyzer (Spectro 320, Instrument Systems, Germany).

### SERS and SEHRS measurements

Surface-enhanced Raman and hyper-Raman spectra of a test analyte (crystal violet) attached to the onion layer were measured at a customized experimental set-up for Raman microscopy using one- and two-photon excitation [23]. The same objective (NA = 1.2) was used for providing the excitation laser and for collecting the scattered light. Placing the sample on a moving stage allows for the collection of SERS and SEHRS images. Two-photon excited hyper-Raman signals were generated by 1064 nm mode-locked Nd:YAG laser excitation (7 ps pulse duration, 76 MHz repetition rate). The second harmonic wave length of this laser at 532 nm was used for one-photon excitation. Applied peak photon flux densities at the samples were 1 × 10^29^ photons·cm^−2^·s^−1^ and 5 × 10^25^ photons·cm^−2^·s^−1^ and collection times were 10 s and 2 s for SEHRS and SERS spectra, respectively. Signal strengths for creating images were determined by reading out the maximum signal of the band at 1175 cm^−1^ and subtracting the signal at 1130 cm^−1^ as background.

## Results and Discussion

**Figure 1:**
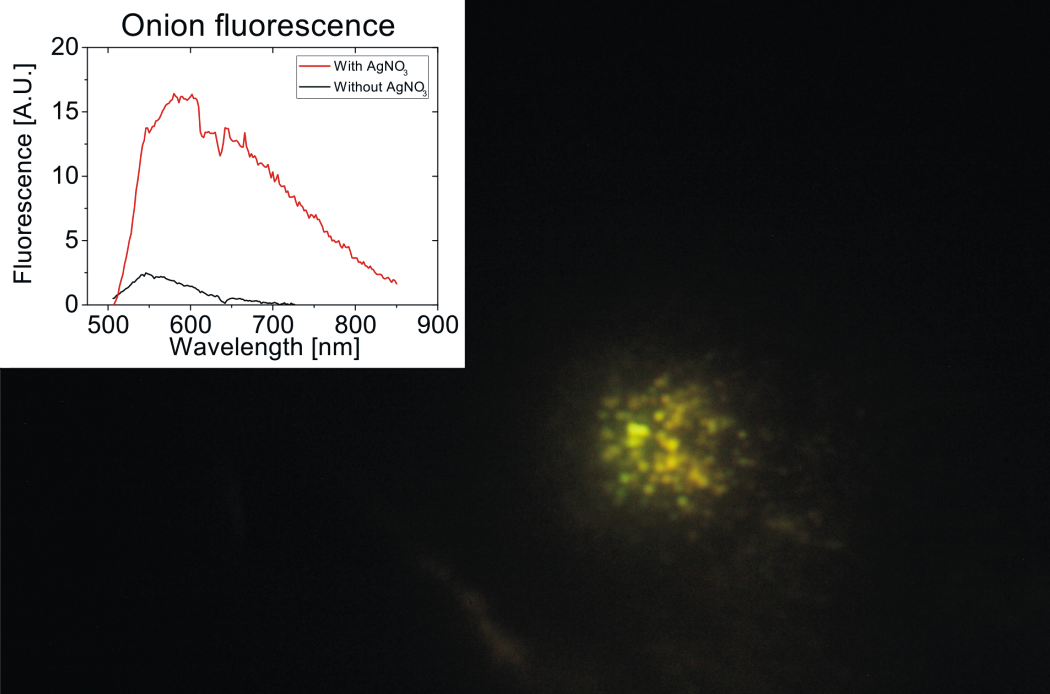
Image and spectrum of multi-color luminescence collected from onion cell layers after incubation with AgNO_3_. The excitation wavelength was at 473 nm provided by a laser diode operated at around 5 mW through a 100× oil immersion objective. The illuminated spot is ca. 10 µm^2^.

Among various plant materials applied for green synthesis of metal nanoparticles, also the use of onion extract for the preparation of gold and silver nanoparticles has been reported [24–25]. In all those studies, onions have been crushed and boiled and finally, onion extract has been employed in the preparation process. Here, we explore the in situ preparation of metal nanoparticles in intact fresh onion cell layers at room temperature. After about 20 h of exposure to AgNO_3_ solution, and a following drying period of 2–3 h, the onion samples appear in a reddish color, compared to their initial whitish color, suggesting the formation of nanoparticles. Additionally, Figure 1 shows a strong luminescence signal in yellow-greenish colors emitted from the onion layers upon excitation at 473 nm.

In general, bright luminescence signals have been discovered as characteristic optical signatures of small silver clusters [26]. The luminescence observed from the onion layer hints to the existence of small silver clusters Ag*_n_*^+^ formed from Ag^+^ available after the initial reduction process of AgNO_3_ [27]. The presence of various small silver clusters in the onion tissue is supported by previous observations reported for the green synthesis of silver nanoparticles using orange extract [19]. There, the same fluorescence signals as shown in Figure 1 have been observed [19]. UV absorption measurements in this study show a band at 270 nm, confirming the existence of Ag_4_^2+^ clusters, which, via intermediate larger clusters, eventually form metallic particles Ag*_n_* [27]. From these small metal particles, plasmonic silver particles grow by coalescence [27–28]. The dark red color of the onion layer described above is an indicator that plasmonic silver nanoparticles have formed, including also the formation of aggregates.

**Figure 2:**
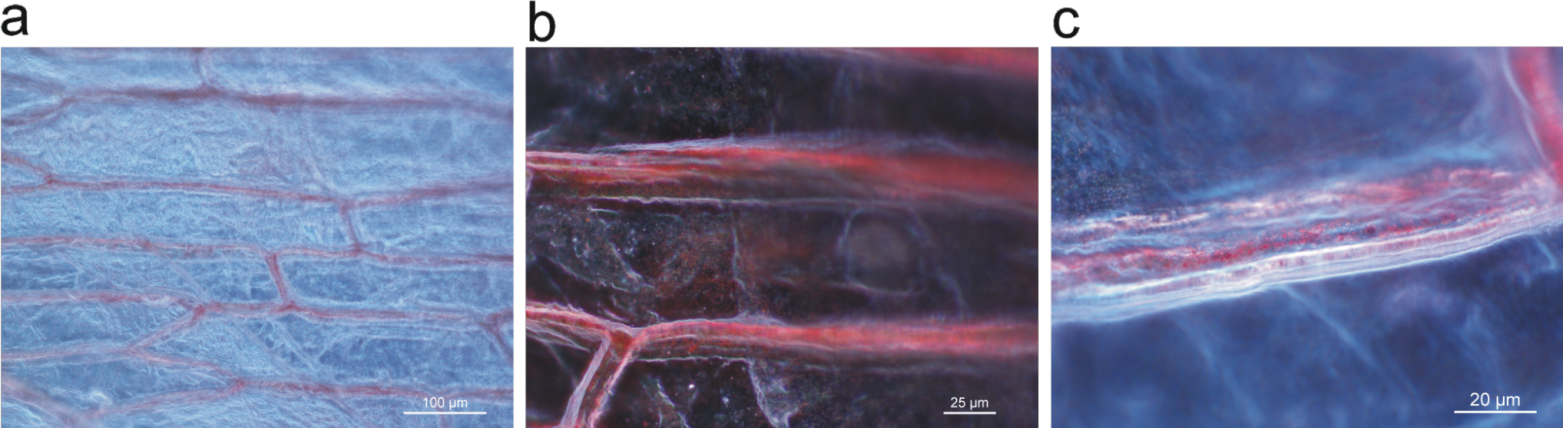
Dark field images of onion cell layers after incubation with AgNO_3_ solution. The magnification is a) 10×, b) 20× and c) 50×, respectively. Scattering of red light originating from silver nanoparticles appears in the extracellular space of the epithelial layer.

Dark field microscopy provides more detailed spatial information about where these silver nanostructures exist. The red glowing of the extracellular matrix of the onion layer in the dark field images shown in Figure 2 is due to the scattered light from silver nanoparticles and their aggregates. The dark field images of the onion cell layer show that the plasmonic silver nanostructures grow preferentially in the extracellular matrix between the epidermal cells of the onion tissue. The luminescence pattern in Figure 1 shows that some silver ions are taken up into the protoplast during the osmotic imbalance when the hypertonic silver salt solution is added, and the small clusters must be stabilized there. In contrast, at the outer cell walls and in the extracellular space a biomolecular environment is provided that enables the templated growth and the stabilization of larger plasmonic nanoparticles. As suggested by uptake studies with other metal ions into epidermal cells, adsorption of the silver to the extracellular matrix is expected to be faster than uptake into the cells [29]. Several molecular candidates can be responsible for the reduction and stabilization of the nanoparticles in these regions, specifically pectin, which is part of the cell wall, and which has been shown to be an efficient reducing and stabilizing agent for the synthesis of silver nanoparticles in several studies [30–32]. Glycoproteins that have a function in cellular adhesion or have enzymatic activity such as alliinase, which is a major protein component of *Allium sp*., provide several possibilities for the reduction and the stabilization of the silver metal nanoparticles.

**Figure 3:**
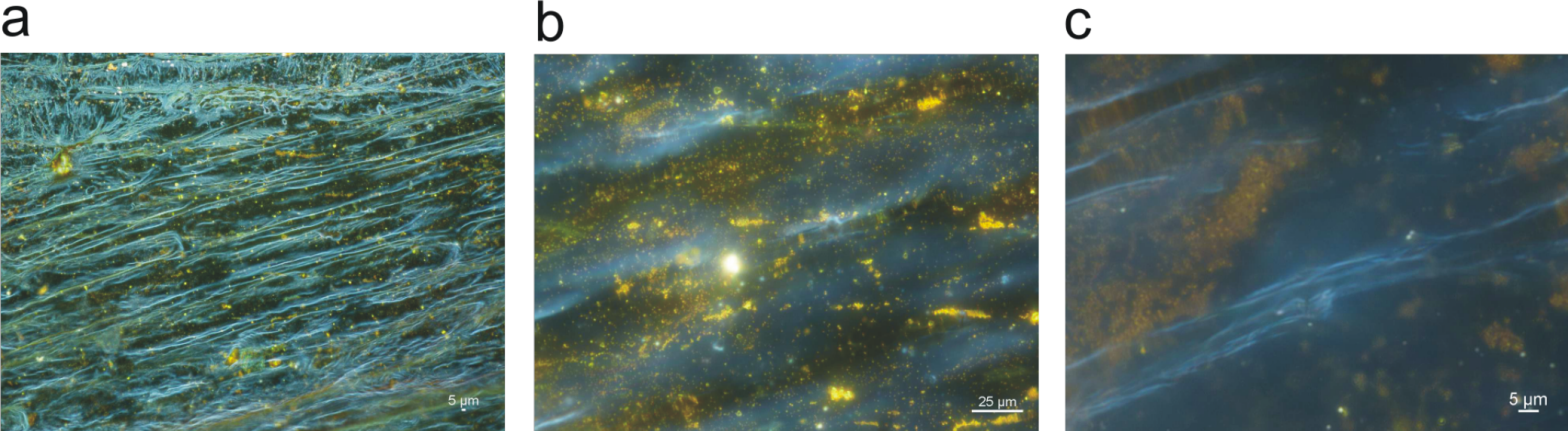
Dark field images of onion layers after incubation with HAuCl_4_ solution, using a similar protocol as in Figure 2. The magnification is a) 10×, b) 20× and c) 50×, respectively. The scattering of yellow light originating from gold nanoparticles appears without any correlation to the cellular structure of the onion layer.

For comparison, Figure 3 shows dark field images of onion cell layers after 20 h of incubation with chloroauric acid. The onion samples incubated in chloroauric acid did not show any change of color after drying. In the dark field micrographs, yellow light scattered from small gold nanoparticles shows that these gold structures are formed only at a few points without any correlation to the cellular structure of the epithelial tissue. A templating effect due to the cellular matrix of the onion layer as it has been observed for silver does not exist for the formation of gold nanoparticles.

Raman scattering experiments performed on onion cell layers exposed to HAuCl_4_ show a strong Raman line at 346 cm^−1^, which exists across the entire sample. This line can be assigned to an Au–Cl stretching vibration [33] and indicates the presence of a large amount of excess gold chloride. This suggests that reduction agents available in the onion layers are obviously not very efficient for the reduction of HAuCl_4_. This is surprising since onion extract was reported to work well for the reduction of HAuCl_4_ [24]. On the other hand, green synthesis involves complex mixtures of molecules with both reducing and stabilizing function, which can indeed be different in plant extracts and in plant tissue, where compartmentalization and spatial separation also play a role. Our experiments indicate that the chemical composition and micromorphological structure in onion epidermal tissue is much more efficient for the reduction of AgNO_3_ than for the reduction of HAuCl_4_.

In the following, we check silver and gold nanostructures grown in onion cell layers regarding their capability as field-enhancing plasmonic structures. SERS tests on onion layer–gold samples as shown for example in Figure 3 using 633 and 785 nm excitation wavelengths and crystal violet (CV) as test analyte resulted in only very weak SERS signals that were collected only from particular places of the sample where gold nanostructures were found (data not shown). The extremely poor SERS signals indicate that the gold nanostructures formed in the onion layers do not support high local fields.

**Figure 4:**
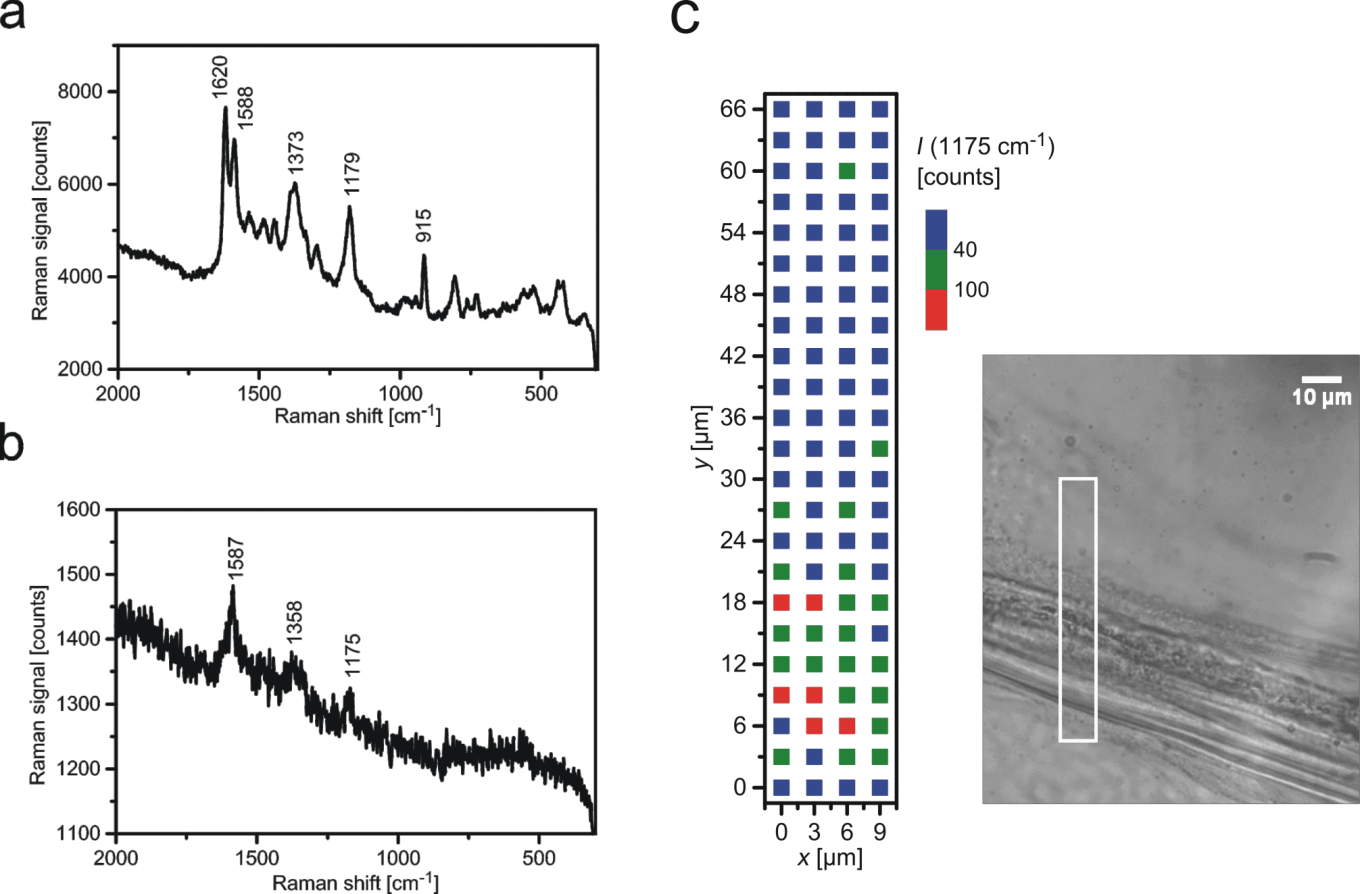
SERS and SEHRS measured from crystal violet attached to an onion cell layer containing plasmonic silver nanostrcutures in the extracellular space. a) SERS spectrum of crystal violet measured using 23 μW pulsed excitation at 532 nm, collection time 2 s*.* b) SEHRS spectrum of crystal violet measured using 1064 nm pulsed excitation; collection time 10 s. About 10^3^ molecules contribute to the obtained SERS and SEHRS signals. c) Two-photon-excited hyper-Raman image collected from the rectangle shown in the bright field picture of the onion layer. The image displays the 1175 cm^−1^ SEHRS band (see spectrum in panel b). The color code represents the signal from lowest (blue) to highest (red).

In contrast, silver nanostructures grown in onion layers are well suited for SERS experiments. Figure 4a shows a typical SERS spectrum collected from CV attached to onion–silver samples. The strongest SERS signals were obtained from the region of extracellular space and cell walls. We did not measure SERS signals that we could ascribe to intrinsic biomolecules present in the plant tissue. This is in accordance with the assumption that mainly carbohydrate species (such as pectin) are present the cell walls and in the extracellular space and with the finding that SERS measurements of these molecules require special functionalization of the silver nanostructures [34]. Furthermore, the intrinsic plant molecules are probably present at much lower concentrations than the test molecule, where about 1000–2000 CV molecules contribute to the observed SERS signals. Moreover, at the applied 532 nm excitation, CV benefits from additional resonance enhancement as a further advantage over colorless biomolecules.

In addition to one-photon-excited SERS we checked the silver nanostructures in onion layers also by two-photon-excited SEHRS. In a hyper-Raman process, two photons are scattered simultaneously and the hyper-Raman spectrum appears shifted relative to the second harmonic of the excitation laser. Since we excited normal SERS using the second harmonic of the 1064 nm SEHRS excitation laser, SERS and SEHRS spectra shown in Figure 4a and Figure 4b, respectively, appear in the same spectral range. Figure 4b shows a typical SEHRS spectrum collected from the same sample. Spectra in Figure 4a and Figure 4b show the well-known Raman and hyper-Raman features of CV [35].

The main enhancement mechanism for SERS and SEHRS are high local fields in the vicinity of metal nanostructures related to resonances between light and surface plasmon modes of these structures [36]. Therefore, SERS or SEHRS can be exploited to probe local optical fields [37]. As mentioned above, its high non-linearity makes SEHRS a very sensitive tool to image spatial variations in local fields and to localize plasmonic nanostructures. Here we perform SEHRS scans across the onion sample. A SEHRS image, shown in Figure 4c, indicates that places displaying highest SEHRS signals correlate with the extracellular space and cell walls in the onion cell layer. SEHRS images support the finding from dark field images and show that plasmonic silver nanoparticles are formed and confined to the extracellular space and cell walls.

## Conclusion

Our studies demonstrate the green synthesis of plasmonic silver nanoparticles in onion layers after incubation with AgNO_3_ solution without any additional reducing or stabilizing chemicals. The plant delivers not only the required chemicals, the extracellular matrix including cell walls in the onion layer also provide a bio-template for growing the plasmonic silver nanostructures. Green silver nanostructures grown in the cell walls and extracellular space are suited for enhancing one- and two-photon-excited surface enhanced Raman and hyper-Raman scattering, respectively. Our studies suggest also the preparation of silver nanoparticles directly inside living plants after the uptake of solutions of metal salts. This would be of particular interest as an efficient method to deliver silver nanoparticles as basic building blocks of SERS nanosensors [38–40] to study chemical compositions and processes inside plants.
